# Drug related problems in admitted geriatric patients: the impact of clinical pharmacist interventions

**DOI:** 10.1186/s12877-020-1413-7

**Published:** 2020-01-13

**Authors:** Berhane Yohannes Hailu, Derebew Fikadu Berhe, Esayas Kebede Gudina, Kidu Gidey, Mestawet Getachew

**Affiliations:** 1 Department of Clinical Pharmacy, School of Pharmacy, College of Health Sciences, Mekelle University, Mekelle, Ethiopia; 2 Department of Pharmacology and Toxicology, College of Health Sciences, Mekelle University, Mekelle, Ethiopia; 3 Department of Internal Medicine, Institute of Health, Jimma University, Jimma, Ethiopia; 4 Department of clinical pharmacy, School of Pharmacy, Institute of Health, Jimma University, Jimma, Ethiopia

**Keywords:** Geriatrics, Drug related problems, Pharmacist interventions

## Abstract

**Background:**

Geriatric patients are at high risk of Drug Related Problems (DRPs) due to multi- morbidity associated polypharmacy, age related physiologic changes, pharmacokinetic and pharmacodynamics alterations. These patients often excluded from premarketing trials that can further increase the occurrence of DRPs. This study aimed to identify drug related problems and determinants in geriatric patients admitted to medical and surgical wards, and to evaluate the impact of clinical pharmacist interventions for treatment optimization.

**Methods:**

A prospective interventional study was conducted among geriatric patients admitted to medical and surgical wards of Jimma University Medical Center from April to July 2017. Clinical pharmacists reviewed patients drug therapy, identified drug related problems and provided interventions. Data were analyzed by using SPSS statistical software version 20.0. Descriptive statistics were performed to determine the proportion of drug related problems. Logistic regression analyses were performed to identify the determinants of drug related problems.

**Results:**

A total of 200 geriatric patients were included in the study. The mean age of the participants was 67.3 years (SD7.3). About 82% of the patients had at least one drug related problems. A total of 380 drug related problems were identified and 670 interventions were provided. For the clinical pharmacist interventions, the prescriber acceptance rate was 91.7%. Significant determinants for drug related problems were polypharmacy (adjusted odds ratio [AOR] = 4.350, 95% C.I: 1.212–9.260, *p* = 0.020) and number of comorbidities (AOR = 1.588, 95% C.I: 1.029–2.450, *p* = 0.037).

**Conclusions:**

Drug related problems were substantially high among geriatric inpatients. Patients with polypharmacy and co-morbidities had a much higher chance of developing DRPs. Hence, special attention is needed to prevent the occurrence of DRPs in these patients. Moreover, clinical pharmacists’ intervention was found to reduce DRPs in geriatric inpatients. The prescriber acceptance rate of clinical pharmacists’ intervention was also substantially high.

## Background

In developing countries including Ethiopia, life expectancy is increasing. This is partly as a result of increased healthcare seeking behavior in the society and increased access to health service [1, 2]. Related to this, population aging has resulted in an increased prevalence of chronic diseases and thus rise in hospitalizations and healthcare costs of older adults [3].

The geriatric population is at high risk of drug related problems (DRPs) due to the age-related pharmacokinetic and pharmacodynamic changes [4]. Furthermore, a higher incidence of drug related problems could result from age associated increased prevalence of multiple chronic diseases, which causes the use of complex therapeutic regimens [5]. According to pharmaceutical care network of Europe (PCNE), *DRP is defined as, “an event or circumstance involving drug therapy that actually or potentially interferes with desired health outcomes”* [6]*.* DRPs are associated with increased healthcare costs and hospital admissions, prolonged hospital stays, reduced quality of life, and increased mortality [7, 8]. Therefore, drug prescribing and use in older patients needs special considerations including avoidance of inappropriate drugs, rational utilization of indicated medications, side effects monitoring, prevention of drug-drug interactions, and evaluation of adherence and patient involvements [9].

The identification, resolution, and prevention of DRPs have been described as a core process of pharmaceutical care. Clinical pharmacists are suitably trained to carry out medication reviews in geriatric patients and have been found to improve the use of high-risk medications and improve the accuracy of medication regimens [10, 11]. In order to resolve DRPs, the cause should be identified and DRPs should be classified appropriately. For such purpose, the classification of DRPs is crucial. There are several classifications for DRPs. However, there is no single standardized classification in the world [12]. The PCNE classification system is commonly practiced and has better usability and internal consistency as it is updated and revised periodically. It is very important for the documentation of DRPs in the pharmaceutical care process [13].

Ethiopia is the second from the top six countries, in which life expectancy and the number of geriatric population is increasing [14] however, there is limited attention for these older patients. Most of the health sciences training programs didn’t include gerontology training in their curriculums and there is no guideline that focuses on geriatric medicine. At the hospital level, geriatric wards are not established for these special populations.

As with other health care services, geriatrics care requires health care professional team work including clinical pharmacists. Experience from developed nations has shown involving clinical pharmacist in patient care resulted in a reduction of DRPs as well as associated costs [15, 16]. Despite the good start up including patient-oriented pharmacy curriculum, clinical pharmacy service is still at the infant stage in Ethiopia. With poor involvement of clinical pharmacists on geriatric care, there is limited information on magnitude of DRPs, determinant factors and clinical pharmacist’s interventions among geriatric patients in hospital set up. Therefore, this study aimed to identify drug related problems and determinants in geriatric patients admitted to medical and surgical wards, and to evaluate the impact of clinical pharmacist interventions for treatment optimization.

## Methods

### Study design and setting

A prospective interventional study was conducted at medical and surgical wards of Jimma University medical center (JUMC). JUMC is the only teaching and referral hospital with 500 beds in the southwestern part of the country located in Jimma town, Southwest Ethiopia, 352 km far from Addis Ababa.

### Study population

All geriatric patients ≥60 years who were admitted for at least 24 h in the medical and surgical wards of JUMC during the study period (01 April 2017 to 31 July 2017) were included. Geriatrics patients were defined as patients age 60 years and older. Although most developed world countries have accepted the chronological age of 65 years as a definition of ‘elderly’, this does not adapt well to the situation in Africa and it can be extended to 60 years and above [17, 18]. Accordingly, we used the cut point of 60 years old in our study. Geriatric patients re-admitted during the study period and patients who refused to participate in the study were excluded.

### Data collection

Patients were interviewed using a standard questionnaire and their respective medical chart was reviewed using data abstraction format. Four MSc clinical pharmacists were involved in the identification, interventions and, documentation of DRPs. DRPs were identified by using the following literature resources such as Ethiopian guidelines, European or American standard guidelines such as, American diabetic association for diabetes, American heart associationguideline for heart failure, American urologic association guideline for benign prostatic hyperplasia, world health organization guideline for surgical site infection prophylaxis and other relevant guidelines for the respective diseases identified. Moreover, adverse drug reactions (ADR) were assessed according to the NaranjoADR probability scale [19]. Micromedex was used to check drug-drug interactions. Pharmaceutical care network Europe (PCNE) DRPs classification system was used to classify and document DRPs [6]. For the identified DRPs, intervention was provided through discussion with individual prescriber immediately. Additionally, recommendations were forwarded during rounds and morning sessions orally and/or with written documents and the prescriber acceptance documented. DRPs which are not accepted were further discussed with a senior physician for confirmation. The acceptance of the recommended interventions was followed to check for their implementations and the status of the intervention was documented.

### Outcome measure

We have two main objectives in the study. The first objective is to measure the prevalence and determinates of DRPs among geriatric population attending surgical and internal medicine wards of JUMC. The second objective of the study is measuring the impact of clinical pharmacy intervention on reducing the number of DRPs and the acceptance rate of clinical pharmacists’ intervention by prescribers.

### Explanatory variables

DRPs were identified by evaluating the appropriateness of drug therapy in terms of indication, dosage, safety, and efficacy. For the identified DRPs, the clinical pharmacists provided interventions. Clinical pharmacist interventions were defined as any action by a clinical pharmacist that directly results in a change of patient management. The Pharmaceutical care network Europe DRPs classification system [6] used in this study have different parts that include: i) The problems(e.g. effect of drug treatment not optimal, unnecessary drug-treatment), ii) causes (e.g., drug dose too low, wrong drug administered, inappropriate timing or dosing intervals), iii) planned interventions (e.g. intervention discussed with prescriber, patient (drug) counselling), iv) acceptance of the intervention proposals (intervention accepted or not accepted) and v) outcome of the intervention (problem solved, partially solved or unsolved). Different interventions were applied for some of the DRPs that resulted in the number of interventions greater than the number of DRPs.

The other variables assessed were (1) socio-demographics (including sex, age, financial status, educational level, residence, marital status, and social support) (2) disease related factors (diagnosis, co-morbidities) and (3) drug/therapy related factorsthat include **(**polypharmacy, class and type of drugs).Poly-pharmacy was considered when the patients were prescribed with five or more drugs together. Patients who had one or more additional disease condition in addition to the main disease were considered as having comorbidity.

### Statistical analyses

EpiData version 4.0.2. for data entry and Statistical Package for Social Science (SPSS) version 20 (IBM SPSS version 20.0 Inc., Chicago, Illinois) were used for data analysis. Categorical variables were expressed in terms of frequencies and percentages. Continuous variables were presented using mean and standard deviation. The proportion of DRPs and prescriber’s acceptance rate of the intervention were reported using these descriptive statistics. Univariable logistic regression analysis and multivariable logistic regression analysis was performed to determine the potential determinant of DRPs. Results were reported as odds ratios (ORs) with 95% CIs. A *p* value of less than 0.05 was considered significant.

## Results

### Socio-demographic characteristics

A total of 200 patients were included in the final analysis. Of this, 135(67.5%) were male, the mean (SD) age was 67.3 (7.5) years, and the majority (68.5%) of the patients were from rural areas. Two third of the patients did not have a formal education and only 36.5% of the patients had a monthly income of ≥800 Ethiopian Birr. Furthermore, 75% of them relied on the caregiver (Table 1).

**Table 1:** The Socio-Demographic Characteristics of geriatric patients admitted from April to July to Medical and Surgical wards of JUMC, Ethiopia, 2017 (*N* = 200)

Demographic characteristics	N (%)
Age, Mean (SD)	67.3 (7.3)
Sex male	135 (67.5)
Marital status	
Married	158 (79)
Widowed	36 (18)
Divorced	6 (3)
Religion	
Christian	50 (25)
Muslim	150 (75)
Educational status	
No formal education	132 (66)
Primary education	55 (27.5)
Secondary education	8 (4)
Tertiary education	5 (2.5)
Residence	
Rural	137 (68.5)
Urban	63 (31.5)
Monthly income	
< 800 birr	127 (63.5)
≥ 800 birr	73 (36.5)
Relies on caregiver (yes)	150 (75%)

### Clinical and medication characteristics

The majority (65. 5%) of the patients were admitted to the medical ward. Participants had an average of 2.20 (SD1.57) clinical conditions and they took an average of 3.90 (SD2.11). The most common medical condition was heart failure (24%) followed by stroke (13%) and benign prostatic hyperplasia (11%). Polypharmacy was present in 35.5% of the patients (Table 2).

**Table 2:** Clinical, and medication related characteristics in geriatric patients admitted from April to July at Medical and Surgical wards of JUMC, Ethiopia, 2017 (*N* = 200)

Characteristics	n (%)
Admission ward	
Medical	131 (65.5)
Surgical	69 (34.5)
Mean number of disease condition per patient, Mean (SD)	2.20 (1.157)
Mean number of medications per patient, Mean (SD)	3.9 (2.108)
Polypharmacy (yes)	71 (35.5)
Disease conditions	
Heart failure	48 (24)
BPH	26 (13)
Stroke	22 (11)
Acute coronary syndrome	13 (6.5)
Acute abdomen	12 (6)
Type II DM	11 (5.5)
Cancer	9 (4.5)
Pneumonia	8 (4)
Surgical site infection	8 (4)
Chronic kidney disease	7 (3.5)
Trauma	6 (3)
Hernia	5 (2.5)
Hematoma	5 (2.5)
Gangrene	5 (2.5)
Upper GI bleeding	5 (2.5)
Others	10 (5)

### Prevalence of drug related problems

A total of 380 DRPs were identified from 81.5% of the study participants. Every patient had an average of 1.90 (SD1.47) DRPs. The most commonly found DRPs belonged to the treatment effectiveness related (effect of drug treatment not optimal, untreated indication, and no effect of drug treatment) with 47.6%, followed by (unnecessary drug treatment, problem with the cost-effectiveness of treatment) 28.2%, and adverse drug event 24.2%. Regarding the number of DRPs, 24.5% had one, 26.5% of patients had two, and 30.5% had three and more DRPs (Table 3). Some examples and description of DRPs were provided in Table 4.

**Table 3:** DRP categories and number of DRPs among geriatric patients admitted from April to July to Medical and Surgical wards of JUMC, Ethiopia, 2017

Total number of DRPs =380	n (%)
Problem domains	
P1: treatment effectiveness (3 categories)	181 (47.6%)
Suboptimal effect of drug treatment	102 (56.4)
Untreated indication	69 (38.1)
No effect of drug treatment	10 (5.5)
P2: treatment safety	92 (24.2%)
Adverse drug event (possibly) occurred	92 (100)
P3: Others	107 (28.2%)
Unnecessary drug treatment	81 (75.7)
Problem with cost effective treatment	26 (24.3)
Number of drug related problems	Frequency (%)
None	37 (18.5)
One	49 (24.5)
Two	53 (26.5)
≥ three	61 (30.5)

**Table 4:** Some examples of DRPs among geriatric patients admitted from April to July to Medical and Surgical wards of JUMC, Ethiopia, 2017

Drug related problem	Category of DRPs	Description
A patient in the age range of 60–70 was admitted to surgical ward with the diagnosis of breast cancer. After one side mastectomy was done, the patient was prescribed with Tamoxifen 20 mg po daily.	Untreated indication	Patient complained severe pain which is 8/10. However, the pain was not treated. Patient has untreated indication and needs morphine 2.5 mg every four hour.
A patient in the age range of 60–70 was admitted to internal medicine ward with the diagnosis of ischemic heart disease (NST Elevated myocardial infarction). The patient was prescribed with Simvastatin 40 mg	Suboptimal effect of drug treatment	High intensity statins are required for patients with NSTEMI. Simvastatin 40 mg is medium intensity statin. Thus, it will have suboptimal effect. Therefore, we recommended Atorvasatin 80 mg.
A patient in the age range of 70–80 admitted with the diagnosis of peripheral arterial disease. After admission the patient prescribed with warfarin 5 mg and Heparin 12,500 IU	No effect of drug treatment (Warfarin and Heparin) Untreated indication	Warfarin and Heparin have no effect on peripheral arterial. Patient needs Atorvastatin and Aspirin to treat his condition.
Known cardiac patient in the age of 80–90 was admitted to internal medicine ward. The patient was taking aspirin and lost around 3 l of blood.	Adverse drug event (possibly) occurred	Aspirin was considered the offensive drug and discontinued for 07 days. The bleeding stopped after aspirin was discontinued. Based on Naranjo scale the bleeding is possibly due to Aspirin.

### Causes of drug related problems

**Fig. 1:**
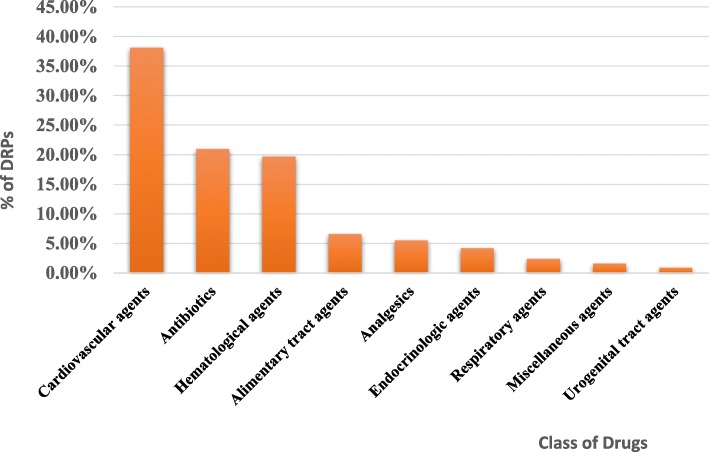
Class of drugs involved in drug related problems in geriatric patients admitted from April to July to medical and surgical wards of JUMC, Ethiopia, 2017

Four hundred sixty six causes of DRPs were identified. Of this, the most common causes of DRPs were inappropriate drug selection (54.1%) followed by inappropriate dose selection (14.6%) and drug use process (12.2%). Among the inappropriate drug selection causes, the presence of new indication for drug treatment (36.1%) was the most common cause followed by no indication for the prescribed drug (20.6%), and inappropriate drug according to guidelines (16.7%) (Table 5). The most frequently involved class of drugs in DRPs were cardiovascular agents (38.1%) followed by antibiotics (21%), and hematological agents (19.7%) (Fig. 1).

**Table 5:** Causes of DRPs identified in geriatric patients admitted from April to July to Medical and Surgical wards of JUMC, Ethiopia, 2017

Cause domain (8 categories) total = 466	n (%)
C1: Drug selection causes	252 (54.1)
New indication for drug treatment	91 (36.1)
No indication for drug	52 (20.6)
Inappropriate drug according to guidelines	42 (16.7)
Contra-indicated	30 (11.9)
Inappropriate duplication of therapeutic	20 (7.9)
Inappropriate combination of drugs, or drugs and food	17 (6.8)
C2: Drug form causes	16 (3.4)
In appropriate drug form	16 (100)
C3: dose selection causes	68 (14.6)
Drug dose too high	46 (67.6)
Drug dose too low	22 (32.4)
C4: treatment duration causes	24 (5.2)
Duration of treatment too long	22 (91.7)
Duration of treatment too short	2 (8.3)
C5: dispensing causes	20 (4.3)
Prescribed drug not available	18 (90)
Prescribing error (necessary information missing)	2 (10)
C6: drug use process causes	57 (12.2)
Drug not administered at all	40 (70.2)
Drug under administered	11 (19.3)
Drug over administered at all	6 (10.5)
C7: patient related causes	22 (4.7)
Patient uses unnecessary drug	7 (31.8)
Patient administered/uses drug in a wrong way	5 (22.7)
Patient cannot afford drug	5 (22.7)
Patient unable to use drug/form as directed	5 (22.7)
C8: other causes	7 (1.5)
No or inappropriate outcome monitoring	7 (100)

### Clinical pharmacist interventions

For identified DRPs, a total of 670 interventions were provided at different levels. Most of the interventions (41%) were provided at the prescriber level, followed by 39.1% at drug level, and 16.1% at patient/caregiver level. At the prescriber level, interventions proposed and discussed with the prescriber were the commonest (82.7%) form of intervention. Out of the interventions carried out at the drug level, the drug stopped and a new drug started was the most common with 30.5% each of them. The prescriber’s acceptance rate was calculated considering the interventions provided at the prescriber level. Accordingly, out of 300 interventions performed at the prescriber level, 275(91.7%) of them were accepted. After the implementation of the interventions, out of the total DRPs, 65.8% of the problems were solved while 27.6% of the problems were not solved (Table 6).

**Table 6:** Intervention, prescriber acceptance rate, and outcome of intervention for DRPs among geriatric patients admitted from April to July to Medical and Surgical wards of JUMC, Ethiopia, 2017

Intervention domain (*N* = 731)	n (%)
I0: No intervention	61 (8.3)
I1: intervention at prescriber level	300 (41.0)
Intervention proposed and discussed with prescriber	248 (82.7)
Prescriber informed only	52 (17.3)
I2: intervention at patient /care giver level	108 (16.1)
Patient drug counseling	84 (77.8)
Spoken to family /care giver	24 (22.2)
I3: intervention at drug level	262 (39.1)
Drug stopped	80 (30.5)
New drug started	80 (30.5)
Dosage changed	40 (15.3)
Drug changed	33 (12.6)
Instruction for use changed	17 (6.5)
Formulation changed	12 (4.6)
I4: other intervention or activity	0
Intervention acceptance domain (*N* = 300)	
A1: intervention accepted at prescriber level	275 (91.7)
Intervention accepted and fully implemented	249 (90.5)
Intervention accepted but not implemented	23 (8.4)
Intervention accepted, implementation unknown	0
Intervention accepted and partially implemented	3 (0.1)
A2: intervention not accepted	25 (8.3)
Intervention not accepted; no agreement	24 (96)
Intervention not accepted not feasible	1 (4)
A3: other (no intervention on acceptance)	0
Problem status domain (*N* = 380)	
O0: problem status unknown	5 (1.3)
O1: problem totally solved	250 (65.8)
O2: problem partially solved	20 (5.3)
O3: problem not solved	105 (27.6)
Lack of coordination of prescriber	56 (53.3)
No possibility to solve problem	47 (44.8)
Lack of coordination of patient	2 (1.9)

### Determinants of drug related problems

In the multivariable logistic regression model, number of disease condition (AOR = 1.588, 95%CI = 1.029–2.450) and polypharmacy (AOR = 3.350, 95% CI = 1.212–9.260) have statically significant association with DRPs. Patients who took five or more medications (polypharmacy) are 3.350 times more likely to have DRPs than those who took less than five medications. Moreover, as clinical condition increases by one unit the likelihood of developing DRPs increases by 58.8% (Table 7).

**Table 7:** Determinants of DRPs among geriatric patients admitted from April to July to medical and surgical wards of JUMC, Ethiopia, 2017

			Univariable logistic regression analysis		Multivariate logistic regression analysis	
Determinants	Yes, n (%)	No, n (%)	*P* value	COR (95% C.I)	*P* value	AOR (95% C.I)
Sex						
Male	108 (66.3)	27 (73 s)		Reference		Reference
Female	55 (33.7)	10 (27)	0.43	1.37 (0.62–3.04)	0.33	1.6 (0.62–4.12)
Marital status						
Married	128 (78.5)	30 (81.1)		Reference		Reference
Divorced	4 (2.5)	2 (5.4)	0.39	0.47 (0.08–2.68)	0.72	1.2 (0.4–3.9)
Widowed	31 (19)	5 (13.5)	0.48	1.45 (0.52–4.04)	0.12	5.7 (0.6–52)
Educational level						
Educated	56 (34.4)	12 (32.4)		Reference		Reference
No formal education	107 (65.6)	25 (67.6)	0.82	1.09 (0.51–2.33)	0.42	1.2 (0.4–3.2)
Reliance on care giver						
No	39 (23.9)	11 (29.7)		Reference		Reference
Yes	124 (76.1)	26 (70.3)	0.463	1.35 (0.61–2.96)	0.55	1.3 (0.5–3.5)
Economic status						
≥ 800 Birr	61 (37.4)	12 (32.4)		Reference		Reference
< 800 Birr	102 (62.6)	25 (67.6)	0.57	0.80 (0.38–1.71)	0.48	1.4 (0.5–3.6)
Residence						
Urban	52 (31.9)	11 (29.7)		Reference		Reference
Rural	111 (68.1)	26 (70.3)	0.79	0.903 (0.415–1.966)	0.47	0.7 (0.3–1.8)
Polypharmacy						
No	97 (59.5)	32 (86.5)		Reference		Reference
Yes	66 (40.5)	5 (13.5)	0.004	4.36 (1.61–11.75)	0.02	3.35 (1.21–9.26)
Comorbidities, mean (SD)	2.20 (1.157)		0.006	1.79 (1.18–2.71)	0.04	1.588 (1.03–2.45)

*COR* Crude odds ratio, *AOR* Adjusted odds ratio, *CI* Confidence Interval

## Discussion

Drug related problems are becoming a major public health concern. Geriatric patients are particularly highly vulnerable to DRPs caused by multiple factors such as polypharmacy and inappropriate prescribing [20]. Identification and prevention of DRPs occurrence in this population is crucial. In our study, DRPs were present in 81.5% of the geriatric patients. This is in line with a study conducted by Ramanath K et al. in internal medicine rural tertiary care hospital of India, which showed an 83.4% prevalence of DRPs [21].

Our study identified an average of 1.90 ± 1.47 DRPs per participant. This is somewhat lower than a study conducted by Chan et al. in Thailand, which showed an average of 2.2 ± 1.6 DRPs per participant [22]. This can be explained by the setting difference. Our study was conducted in hospitalized patients where frequents patients visit by senior physicians and residents were applicable whereas the study in Thailand was conducted on outpatients where patients were less likely to meet senior physicians frequently. More than half (54.1%) of the DRPs resulted from inappropriate drug selection. Among the causes of inappropriate drug selection, having a new indication for drug treatment accounted for a higher proportion (36.1%). This is in line with an interventional study done in Belgium in geriatric wards, which reported the most frequent DRPs was an underuse of medications [23].

After the identification and characterization of DRPs, interventions were proposed by the clinical pharmacists. Interventions were provided at prescriber level, at patient /caregiver level, and at drug level. The physician’s acceptance rate of the clinical pharmacist’s recommendations was determined based on the intervention acceptance rate at the prescriber level. Accordingly, the prescriber acceptance rate was high (91.7%). This is in line with a study conducted in Belgium (87.8%) [23]. This finding has important implications for participating clinical pharmacists as part of the multidisciplinary team, can facilitate the identification of DRPs among geriatric patients and inform physicians to resolve the problems.

Another purpose of our study was to identify the determinants of DRPS. We found that patients with polypharmacy were more likely to develop DRPs than without polypharmacy. In accordance with the present results, previous studies [23, 24] have demonstrated that polypharmacy was a major determinant for the occurrence of DRPs. The observed association of polypharmacy with DRPs in geriatric patients could be as a result of increased health-care costs for the multiple medications, drug-interactions, disability/cognitive impairment, noncompliance to medications, increased risk of adverse drug events, falls and fractures, malnutrition, and functional status decline [3, 25]. We also found that patients having one or more co-morbidities had more DRPs. This finding is in agreement with Tegegneet al [26], the finding which showed patients with comorbidities were at high risk of DRPS.

After the implementation of the clinical pharmacists’ interventions, the status of DRPs was determined. The interventions solved around two-third (65.8%) of the existed drug related problems. However, around 27.6% of the DRPs remained unsolved as a result of lack of coordination of prescribers, no possibility to solve the DRPs and lack of coordination of the patients. These results are consistent with those of other studies which were able to solve 58.9–68.3% of identified drug related problems [23].

## Conclusion

Drug related problems were substantially high among geriatric inpatients. Patients with polypharmacy and co-morbidities had a much higher chance of developing DRPs. Hence, special attention is needed to prevent the occurrence of DRPs in these patients. Moreover, clinical pharmacists’ intervention was found to reduce DRPs in geriatric inpatients. The prescriber acceptance rate of clinical pharmacists’ intervention was also substantially high.

## Data Availability

The dataset analyzed during the current study is available from the corresponding author upon reasonable request.

## Abbreviations

ADR: Adverse drug reaction
AOR: Adjusted odds ratio
CI: Confidence interval
COR: Crude odds ratio
DRPs: Drug related problems
JUMC: Jimma university medical center
PCNE: Pharmaceutical care network europe
SD: Standard deviation
WHO: World health organization
